# Chloroplastic pentatricopeptide repeat proteins (PPR) in albino plantlets of *Agave angustifolia* Haw. reveal unexpected behavior

**DOI:** 10.1186/s12870-022-03742-2

**Published:** 2022-07-19

**Authors:** M. Andrade-Marcial, R. Pacheco-Arjona, E. Góngora-Castillo, C. De-la-Peña

**Affiliations:** 1 Unidad de Biotecnología, Centro de Investigación Científica de Yucatán, Calle 43 No. 130 x 32 y 34. Col. Chuburná de Hidalgo, 97205 Mérida, Yucatán Mexico; 2 Facultad de Medicina Veterinaria y Zootecnia, Consejo Nacional de Ciencia y Tecnología- Universidad Autónoma de Yucatán, Mérida, Mexico; 3 Consejo Nacional de Ciencia y Tecnología-Unidad De Biotecnología, Centro de Investigación Científica de Yucatán, Calle 43 No. 130 x 32 y 34. Col. Chuburná de Hidalgo, 97205 Mérida, Yucatán Mexico

**Keywords:** Agave, Chloroplast, Retro-anterograde communication

## Abstract

**Background:**

Pentatricopeptide repeat (PPR) proteins play an essential role in the post-transcriptional regulation of genes in plastid genomes. Although important advances have been made in understanding the functions of these genes, there is little information available on chloroplastic *PPR* genes in non-model plants and less in plants without chloroplasts. In the present study, a comprehensive and multifactorial bioinformatic strategy was applied to search for putative *PPR* genes in the foliar and meristematic tissues of green and albino plantlets of the non-model plant *Agave angustifolia* Haw.

**Results:**

A total of 1581 *PPR* transcripts were identified, of which 282 were chloroplastic. Leaf tissue in the albino plantlets showed the highest levels of expression of chloroplastic *PPR*s. The search for hypothetical targets of 12 *PPR* sequences in the chloroplast genes of *A. angustifolia* revealed their action on transcripts related to ribosomes and translation, photosystems, ATP synthase, plastid-encoded RNA polymerase and RuBisCO.

**Conclusions:**

Our results suggest that the expression of PPR genes depends on the state of cell differentiation and plastid development. In the case of the albino leaf tissue, which lacks functional chloroplasts, it is possible that anterograde and retrograde signaling networks are severely compromised, leading to a compensatory anterograde response characterized by an increase in the expression of *PPR* genes.

**Supplementary Information:**

The online version contains supplementary material available at 10.1186/s12870-022-03742-2.

## Background

Photosynthetic organisms are essential to supporting life on our planet due to their ability to capture energy from sunlight, providing most of the atmospheric oxygen and key chemical compounds [1, 2]. In the context of these processes, the main eukaryotic subcellular component involved is the chloroplast, an organelle of endosymbiotic origin [3]. The chloroplast in higher plants is part of a diverse group of interconvertible organelles known as plastids. Even though each plastid type exhibits specific metabolic functions in the cell, all of them derive from proplastids, which are characterized by being undifferentiated colorless plastids in cell meristems [4, 5]. Although the chloroplast is a semi-autonomous organelle, the majority of the genes necessary to ensure its complete biogenesis do not reside in its plastome [6, 7]. Around 95% of chloroplastic proteins are encoded from the cell nucleus [8]. Within the wide range of proteins encoded by nuclear genes, those that carry out functions related to regulating RNA metabolism in the chloroplast are considered crucial elements for chloroplast biogenesis and development [9]. Within this group of proteins known as the nucleus-encoded RNA-binding proteins (RBPs) [9] are the mitochondrial transcription termination factor (mTERF) proteins [10], DEAD-Box RNA Helicases (RHs) [11], chloroplast ribonucleoproteins (cpRNPs) [12], pentatricopeptide repeat (PPR) proteins [13], and others. Within the variety of RBPs, PPR proteins are considered one of the most important players in post-transcriptional processes [14, 15].

PPR proteins constitute one of the most numerous eukaryotic gene families in plants, with 400–600 members in terrestrial plant genomes [16, 17]. PPR proteins are structurally characterized by presenting tandem arrays of a degenerate motif of ~ 35 amino acids [18]. This tandem array is made up of 2 to 26 copies of the PPR motifs reported (P, P1, L1, S1, P2, L2, S2, SS, E1 and E2) as well as other domains such as DYW or the SMR [16, 19, 20].

The members of the PPR family have been classified into two subfamilies based on the type of motifs they have. Proteins of the P subfamily have copies only of the canonical P motif. Members of the PLS subfamily exhibit an array of tandem motifs represented by the triad of motifs, P1L1S1 [16, 21, 22]. In addition, members of the PLS subfamily have at their C terminal a combination of motifs that groups them into classes: PLS, E1, E2, E+ and DYW [20]. At a functional level, members of the P subfamily perform functions related to RNA metabolism. These include RNA endonuclease activity, transcript stability, splicing, and translation regulation [23–26]. On the other hand, DYW members of the PLS subfamily with a cytidine-deaminase-like signature have been mainly connected to RNA editing functions [27, 28].

*PPR* genes have central roles in organelle biogenesis and development. For instance, *ppr* knock-out mutant plants frequently have photosynthetic dysfunctions such as low levels of chlorophyll and carotenoids [29], alterations in the conformation of photosystems I and II (PSI and PSII) [25], partial or total decrease in photosynthetic activity [24], increase in the accumulation of reactive oxygen species [30], damage in the chloroplast ribosome biogenesis [31], delayed embryo development [32], lethality of seedlings in early stages of development and abnormal responses to stress [30, 33]. These biochemical and structural disruptions have a direct impact on plant phenotype, as in most cases an albino or pale-green phenotype appears [9].

In recent decades, an attempt to elucidate the factors that could determine the appearance and maintenance of albino phenotypes has been made; however, there is a gap in knowledge about the role of *PPR* genes in a plant without functional chloroplasts (albino). Factors such as the cultivar [34], environmental conditions [35, 36], growth regulators [37, 38], incompatibility between the nuclear and plastid genomes [39], alterations in plastid DNA [40] or alterations in chlorophyll biosynthesis pathways [41] have been proposed as causes of the emergence of albino phenotypes [42]. However, the current approach to studying *PPR* genes has been directed toward the structural and functional description of individual genes in non-albino model plants without a deep understanding of PPR functions. In this study, we present an integrated and novel strategy designed to find and identify chloroplastic *PPR* genes from transcriptome data. Taking into consideration that *PPR* genes are key regulators in chloroplast biogenesis and that the albino somaclonal variant lacks this organelle, knowing the transcriptional behavior of chloroplastic *PPR* genes in the albino plantlet would provide the first clues about their role and possible targets in plants.

## Results

### Putative PPR sequences in the *A. angustifolia* transcriptome

**Fig. 1:**
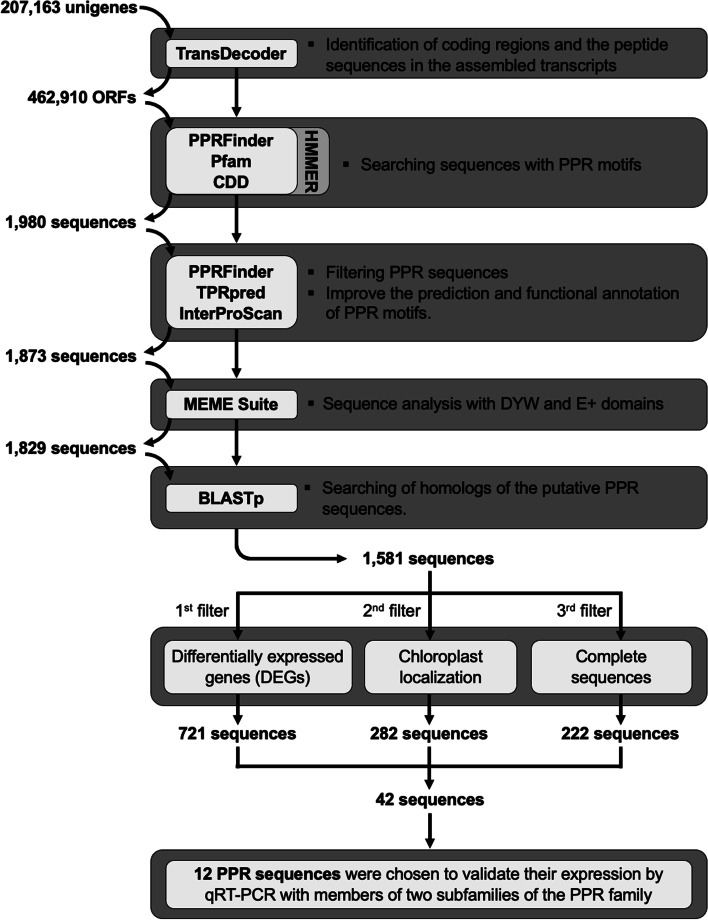
Schematic representation of the bioinformatic pipeline followed in the search for the putative PPR sequences

Triplicate RNA samples from green leaf (GL), albino leaf (AL), green meristem (GM), and albino meristem (AM) of *A. angustifolia* (Additional file 1: Fig. S1) were subjected to Illumina-based sequencing. De novo transcriptome assembly generated 270,163 unigenes, of which 18,829 were differentially expressed (data not shown). The massive identification of sequences harboring PPR motifs was performed in the 462,910 open reading frames (ORFs) predicted from the *A. angustifolia* transcriptome using TransDecoder. The strategy followed here for the search and identification of PPR motifs using PPRFinder, Pfam and CDD’s profiles is summarized in Fig. 1. The analysis with PPRFinder identified PPR motifs distributed in 3089 sequences. On the other hand, the analysis performed with Pfam and CDD profiles identified PPR motifs in 2794 and 2920 sequences, respectively. Thus, the three different strategies helped to identify PPR motifs in a total of 3232 sequences (Additional file 1: Fig. S2A). However, 142 sequences identified with Pfam and CDD profiles were discarded because they did not show a classic array of PPR motifs. This step reduced the number of putative PPR sequences from 3282 to 3090 for downstream analyses (Fig. 1).

The 3090 PPR sequences were analyzed to filter out redundant motifs, and 2095 sequences had an array of motifs similar to that of a classic PPR protein. Of these, 222 sequences were candidates to be joined due to a structural continuity in their tandem array of PPR motifs, given a total of 1980 PPR putative sequences. Additionally, these sequences were analyzed with TPRpred and InterProScan to improve the prediction of PPR motifs (Fig. 1). TPRpred identified PPR motifs in 1904 sequences that do not belong to other protein families with solenoid-type repeats. InterProScan identified 1782, 1754 and 1930 sequences with PPR motifs using the TIGRFAM, PROSITE and Pfam’s profiles, respectively (Additional file 1: Fig. S2B). TPRpred and InterProScan reported a small group of sequences without PPR motifs. However, PPRFinder previously classified these sequences as part of the PLS subfamily, specifically of the E2, E+ and DYW classes. In summary, TPRpred and InterProScan appear to have a low capacity to detect sequences with the DYW domain and variants of the classic PPR motif. Finally, although a 222 ORFs merge is a suggestion of the designers of the PPRFinder code, these sequences were discarded due to their hypothetical structure. Therefore, only 1873 sequences that contain a single ORF were retained for downstream analyses (Fig. 1).

### Sequence analysis with DYW and E + domains

An in-depth analysis was performed with the MEME suite to analyze the structure of the DYW domains in the sequences of the DYW and E+ classes (Fig. 1). Out of 1873 sequences, 231 had DYW domains and 86 had truncated DYW domains. The analysis of the 231 sequences with the DYW domain allowed the identification of three conserved regions: the PG box, the active site and the C-terminal (Additional file 1: Fig. S3). The analysis of each conserved region, along with the alignment of these sequences (Additional file 1: Fig. S4), showed that 23 sequences were individual DYW domains lacking the PG box region and other PPR motifs; these were, therefore, discarded from downstream analyses. Sequences of the E+ class showed an incomplete DYW domain; therefore, only the sequences with an arrangement of motifs at the *N*-terminal and an E+ domain with at least the PG box region were retained for downstream analysis. The alignments of the 86 sequences of the E+ class (Additional file 1: Fig. S5) along with FIMO analysis showed that 65 of the sequences contain a PG region. The remaining 21 sequences were discarded due to lacking motifs at the *N*-terminal and PG conserved region, reducing the total number of sequences from 1873 to 1829 (Fig. 1).

### Functional annotation and structural classification of PPR sequences

In order to identify the closest homolog of the 1829 putative PPR sequences, a local search was performed against a filtered file of plant PPR sequences downloaded from RefSeq (Fig. 1). The results revealed that 75% of the sequences (1389) had the best hits with other PPR sequences from monocot species such as *Asparagus officinalis* (39.41%), *Elaeis guineensis* (26.44%), *Phoenix dactylifera* (12.46%) and *Dendrobium catenatum* (6.45%) (Additional file 1: Fig. S6). A total of 1581 sequences that showed structural PPR motifs and an identity percentage equal to or greater than 50% were retained and deposited at NCBI in the nucleotide database under accession numbers OM156485 - OM158065.

**Fig. 2:**
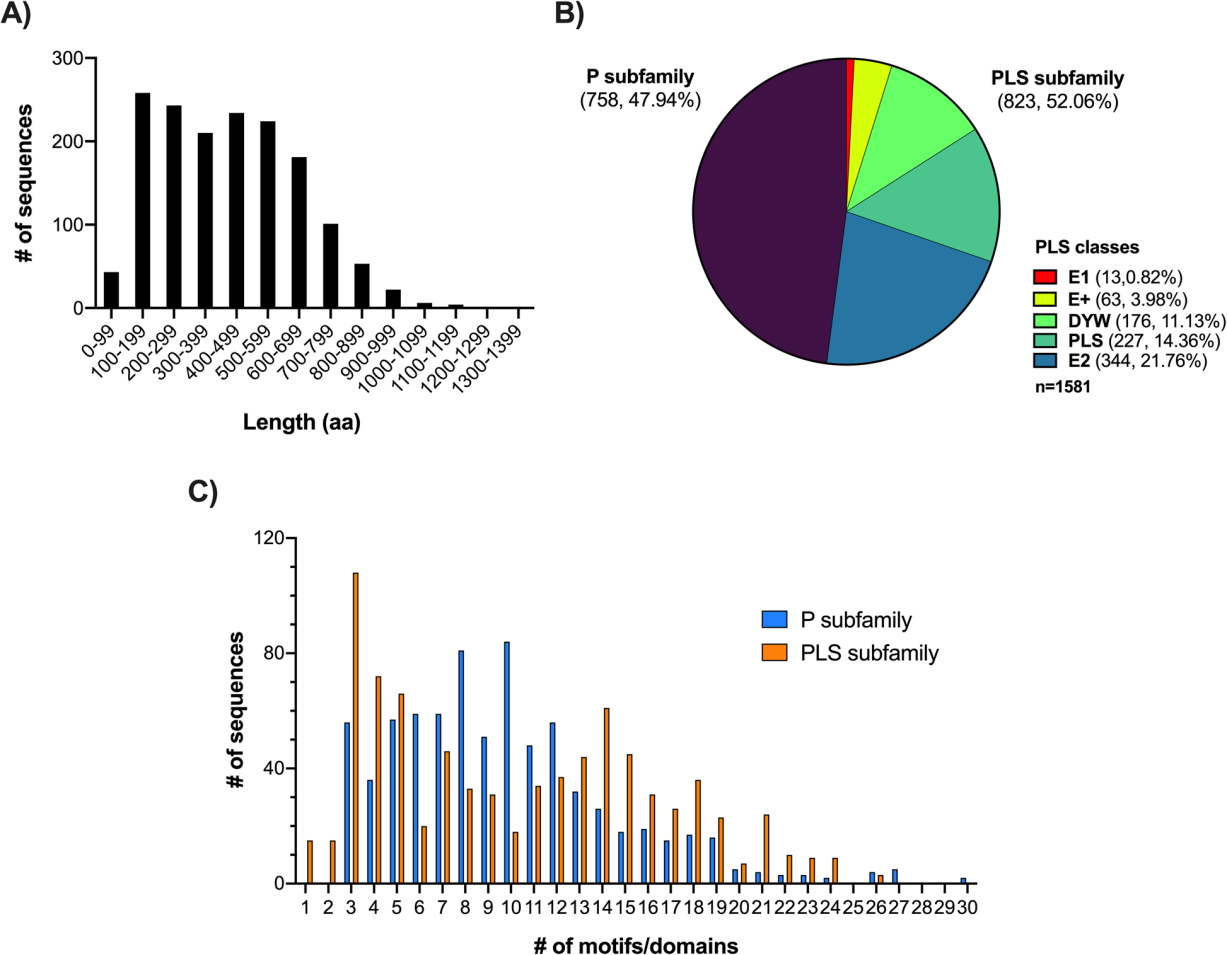
Structural characteristics of the 1581 putative PPR sequences identified in *A. angustifolia.* **A** Frequency of the length in amino acid residues of the putative PPR sequences. **B** Pie chart showing the different subfamilies and classes into which the PPR family is subdivided as well as the number of putative PPR sequences that each one houses. **C** Frequency of the number of PPR motifs detected per sequence and separated according to the P and PLS subfamilies. The nucleotide sequence data of the 1581 *PPR*s for this study are deposited at NCBI in the nucleotide database under accession numbers OM156485 - OM158065

The 1581 putative PPR sequences ranged in length from 58 to 1370 amino acid (AA) residues, with an average length of 427 AA (Fig. 2A). Furthermore, 758 (47.94%) and 823 (52.06%) sequences were grouped within the P and PLS subfamilies, respectively. Within this last subfamily, the E2 and PLS classes were the ones that hosted the highest number of sequences, with 344 (21.76%) and 227 (14.36%), respectively, followed by DYW with 176 (11.13%), E+ with 63 (3.98%) and E1 with 13 (0.82%) (Fig. 2B). The number of PPR motifs per sequence ranged from 2 to 30 (an exception is the case of sequences with a single DYW domain) with an average of 10 motifs per sequence. In the P subfamily it was common to observe sequences with 3–12 PPR motifs and 3–14 motifs from the PLS subfamily (Fig. 2C). More information related to the structural characteristics of these PPR sequences can be found in Additional file 2: Table S1.

### *PPR* transcripts differentially expressed in the transcriptome of *A. angustifolia*

**Fig. 3:**
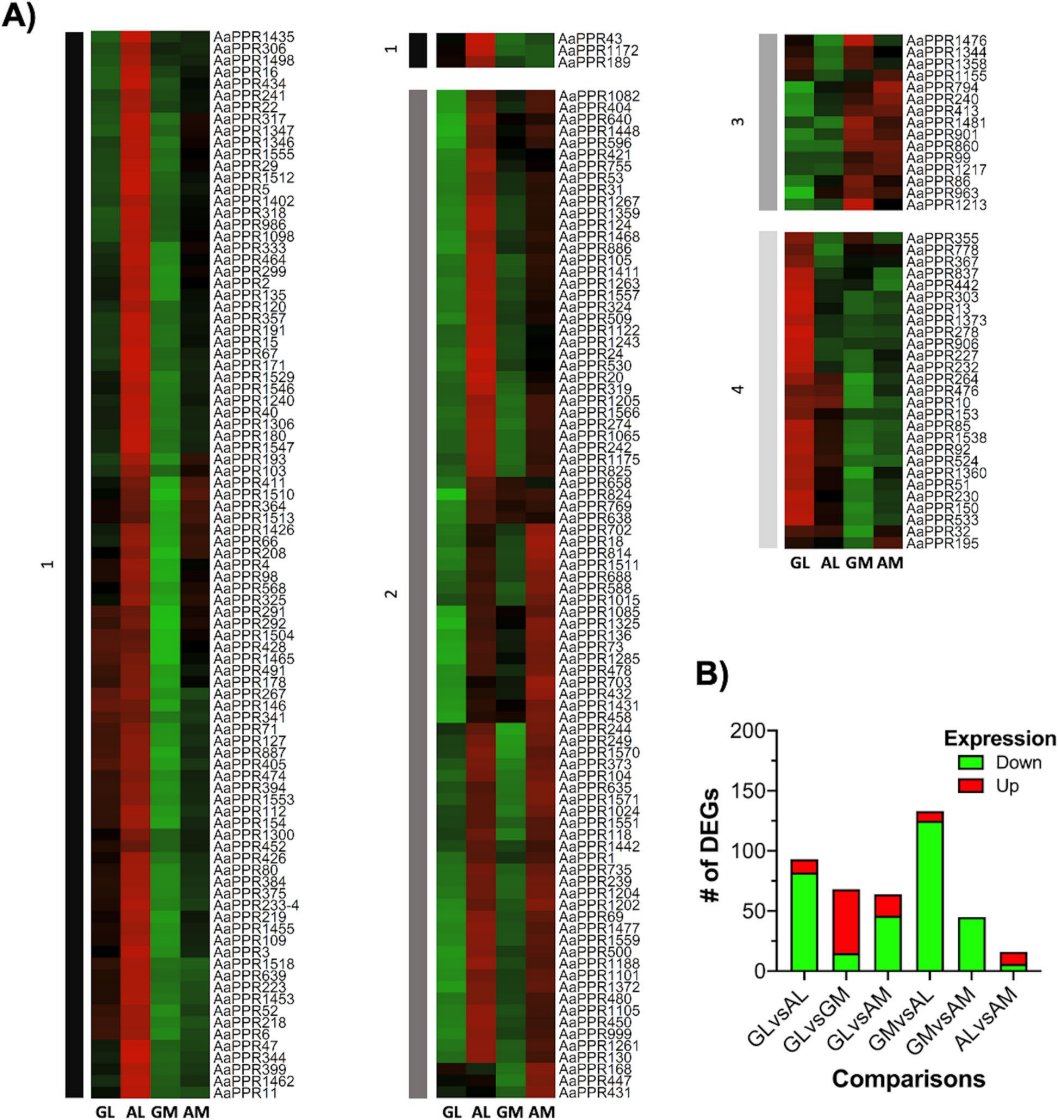
Differentially expressed *PPR* transcripts identified in the transcriptomic analysis of *A. angustifolia*. **A** Heatmap representing the expression profiles of the 222 *PPR* transcripts differentially expressed in the GL, GM, AL and AM tissues of *A. angustifolia*. The four tissues studied are shown at the bottom of the figure, and the transcript identifiers are on the right. Clustering was applied in the heatmap in order to group the transcripts according to their expression levels. The clusters generated are indicated with bars and numbers on the left (1–4). **B** Number of differentially expressed *PPR* transcripts in each of the six tissue pair comparisons studied are shown (GL vs GM, GL vs AL, GL vs AM, GM vs AL, GM vs AM and AL vs AM). In each comparison, the first tissue is the reference to indicate whether the genes are up-regulated (indicated by red bars) or down-regulated (indicated by green bars). The numbers within each bar indicate the number of up- or down-regulated transcripts. GL: leaf from green plantlet, GM: meristem from green plantlet, AL: leaf from albino plantlet, AM: meristem from albino plantlet. Up: up-regulated genes, Down: down-regulated genes

From the 1581 putative PPR sequences found in the transcriptome (GenBank-NCBI, accession number OM156485 - OM158065), a total of 222 were identified as differentially expressed transcripts in the six comparisons between pairs of tissues (GL vs AL, GL vs GM, GL vs AM, GM vs AL, GM vs AM, and AL vs AM) (Fig. 1). The expression profiles of these 222 *PPR* transcripts in the four tissues evaluated (GL, AL, GM and AL) were plotted in a heatmap (Fig. 3A). The expression profiles revealed that in AL tissue and to a lesser extent in AM, a high percentage of *PPR* transcripts is overexpressed with respect to GL and GM tissues, respectively. The expression levels of most *PPR* in GM tissue are the lowest compared to the rest of the tissues (Fig. 3A).

Comparisons between tissues from different phenotypes (GL vs AL, GL vs AM, GM vs AL, and GM vs AM) showed that 88, 71, 93, and 100% of the *PPR* transcripts were down-regulated in green tissues, respectively. The number of *PPR* transcripts up- and down-regulated in each tissue pair comparison is summarized in Fig. 3B. In the comparisons between tissues of the same phenotype, GL vs GM and AL vs AM, the comparison between GL and GM had the highest percentage of overexpressed *PPR* transcripts in leaf tissue (around 78%). On the other hand, AL vs AM was the comparison that showed the lowest number of differentially expressed transcripts, with only 16 (Fig. 3B). Therefore, these results obtained from the transcriptomic analysis (GenBank-NCBI, accession number OM156485 - OM158065) suggest that there is a greater number of *PPR* transcripts overexpressed in tissues of plantlets of *A. angustifolia* with an albino phenotype compared to those with a green phenotype.

### Selection of *PPR* transcripts for validation by qRT-PCR

**Fig. 4:**
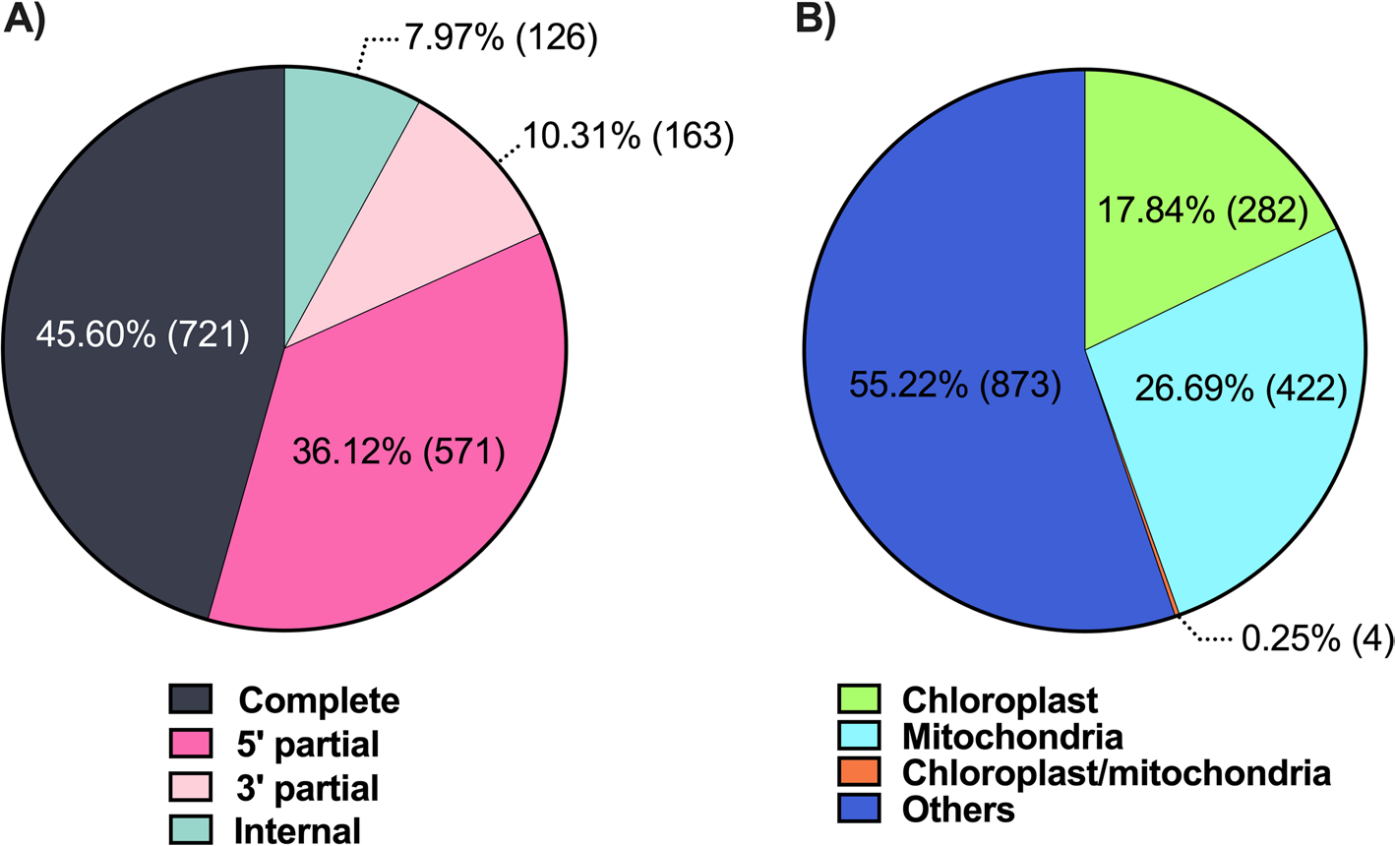
Criteria of selection of PPR sequences for their validation by qRT-PCR. **A** Pie chart showing the status of the identified ORFs based on the results of the Transdecoder software. **B** Pie chart showing the hypothetical subcellular location of putative PPR sequences based on information from their RefSeq orthologs identified by BLASTp analysis. The number of sequences in each category is indicated in parentheses

**Fig. 5:**
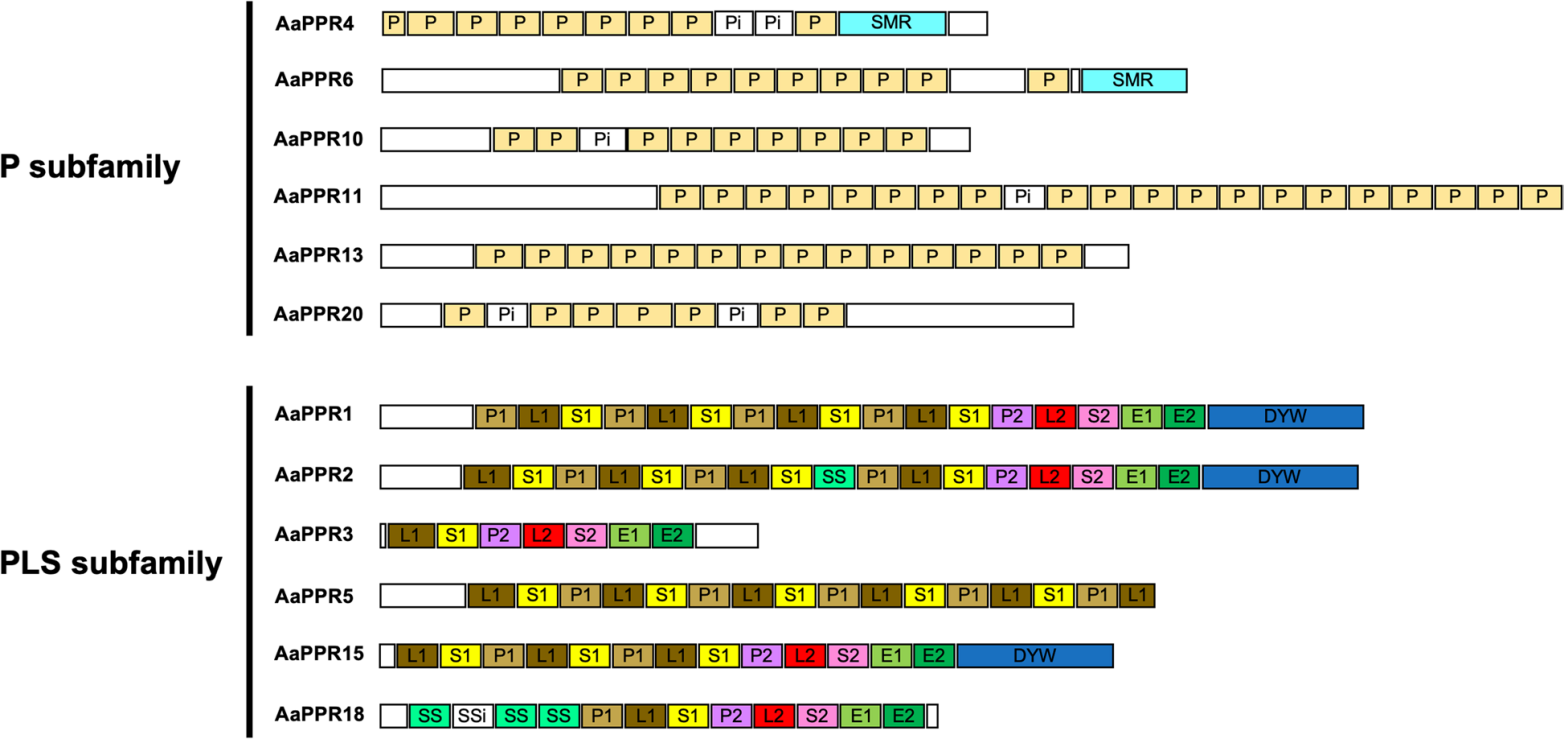
Hypothetical structure of twelve chloroplastic *PPR* transcripts selected for validation by qRT-PCR. Diagram showing the distribution of the PPR motifs/domains identified in each of the proteins encoded by the 12 selected *PPR* transcripts. The PPR motifs identified are represented by boxes of different colors with their respective names. The inferred motifs are indicated with an “i” after the corresponding motif name

The criteria established for the selection of a set of sequences for its validation by qRT-PCR revealed that of the 1581 PPR sequences, a total of 721 sequences (45.60%) were found to have a complete ORF (Fig. 4A), and 282 sequences (17.84%) had an orthologue with a chloroplastic site of action (Fig. 4B). Additionally, the 222 differentially expressed transcripts (14.04%) according to the RNA-seq data were also considered. Only 42 PPR sequences fulfilled the three previous criteria (Fig. 1). Of these 42 sequences, a group of twelve were selected to be validated by qRT-PCR. This selection was made considering a balanced representation of the two subfamilies into which the PPR family is subdivided. *AaPPR1*, *AaPPR2*, *AaPPR3*, *AaPPR5*, *AaPPR15* and *AaPPR18* were the selected transcripts of the PLS subfamily. From the P subfamily, *AaPPR4*, *AaPPR6*, *AaPPR10*, *AaPPR11*, *AaPPR13* and *AaPPR20* transcripts were chosen. Figure 5 shows a structural scale representation of the twelve putative *PPR* transcripts grouped according to the subfamilies/classes to which they were classified.

**Fig. 6:**
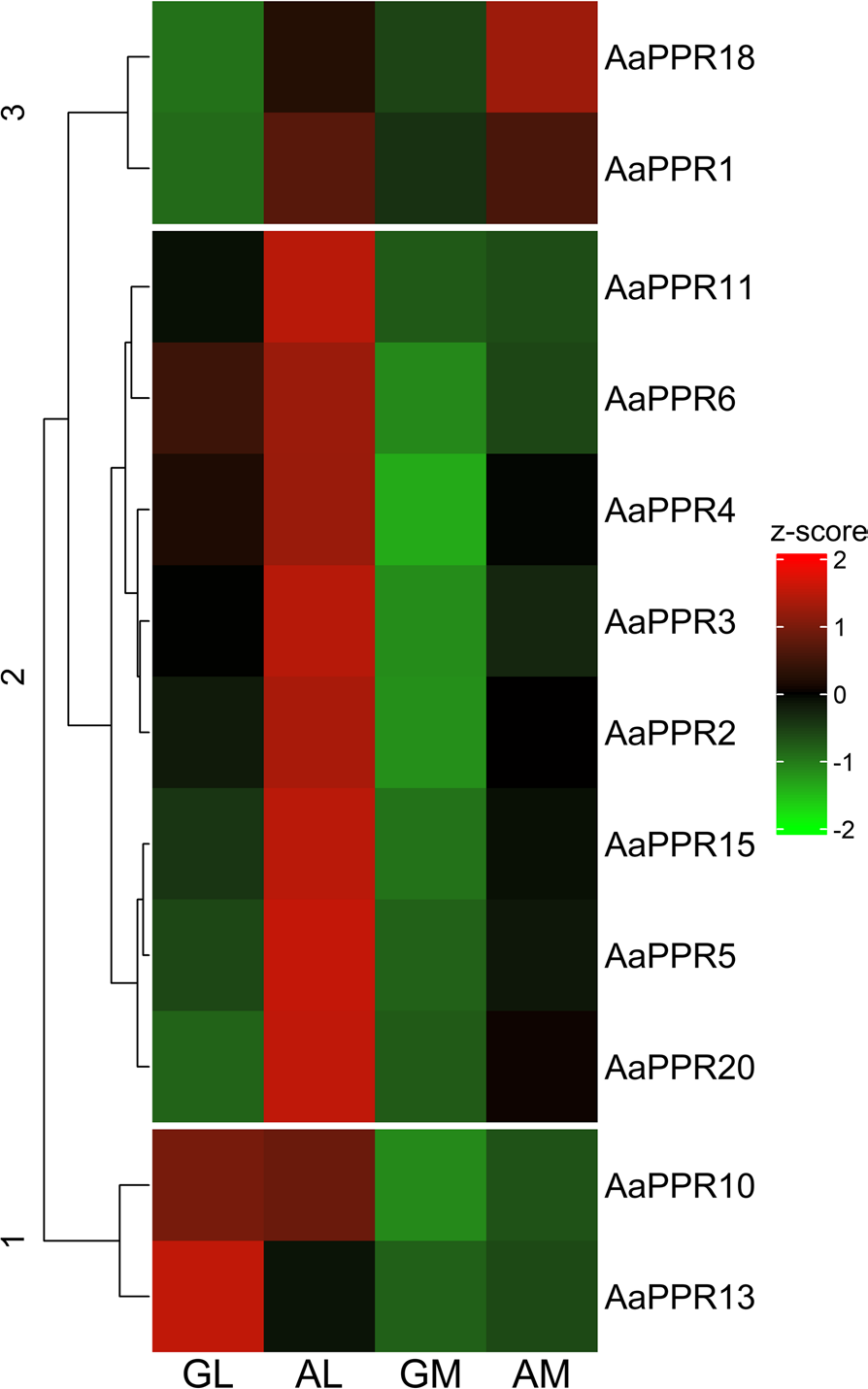
Expression profiles of twelve chloroplastic *PPR* transcripts in *A. angustifolia* obtained from RNA-seq data. The four tissues studied are shown at the bottom of the figure, with the gene identifiers on the right. Clustering was applied in the heatmap in order to group the transcripts according to their expression levels. The clusters generated are indicated by the numbers on the left (1–3). GL: leaf from green plantlet, GM: meristem from green plantlet, AL: leaf from albino plantlet, AM: meristem from albino plantlet

The expression profiles of the 12 selected *PPR* transcripts obtained from the RNA-seq data were represented in a heatmap (Fig. 6). Moreover, a summary of the Log2 Fold change of these genes in each tissue comparison is presented in Additional file 2: Table S2. Differential expression analysis performed on RNA-seq data revealed the following results: in the comparison of GL vs AL, the transcripts *AaPPR1*, *AaPPR5*, *AaPPR15*, *AaPPR18* and *AaPPR20* were over-expressed in AL. In GL vs GM, the transcripts *AaPPR2*, *AaPPR3*, *AaPPR4*, *AaPPR6*, *AaPPR10* and *AaPPR13* were over-expressed in GL, while only *AaPPR1* was over-expressed in GM. In GL vs AM, the transcripts *AaPPR1*, *AaPPR18* and *AaPPR20* were over-expressed in AM, and *AaPPR13* was over-expressed in GL. The comparison between meristems (GM vs AM) showed that *AaPPR2*, *AaPPR3*, *AaPPR4*, *AaPPR18* and *AaPPR20* were over-expressed in AM. In the comparison of GM vs AL, the transcripts *AaPPR1*, *AaPPR2*, *AaPPR3*, *AaPPR4*, *AaPPR5*, *AaPPR6*, *AaPPR10*, *AaPPR11* and *AaPPR20* were over-expressed in AL. Finally, in the comparison between AL and AM, there was no difference in the expression of *PPR* transcripts. In summary, RNA-seq data shows that there is a tendency for these 12 *PPR* transcripts (*AaPPR1*, *AaPPR2*, *AaPPR3*, *AaPPR4*, *AaPPR5*, *AaPPR6*, *AaPPR10*, *AaPPR11*, *AaPPR13*, *AaPPR15*, *AaPPR18* and *AaPPR20*) to be more expressed in leaf tissue than in meristematic tissue, and this is much more evident in AL.

### Expression analysis of *PPR* transcripts by qRT-PCR

**Fig. 7:**
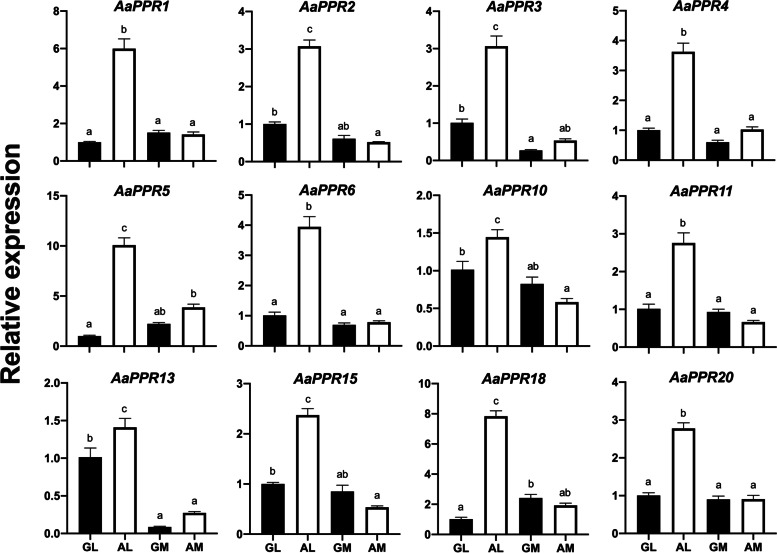
Validation of twelve chloroplastic *PPR* transcripts evaluated in *A. angustifolia* by qRT-PCR. Expression levels for each transcript were normalized using 2–ΔΔCT, taking the GL tissue as the reference expression level. The values of the means and the standard error are represented in each graph. The bars with different letters indicate significant differences between tissues according to Tukey’s test (*p* < 0.05). GL: leaf from green plantlet, GM: meristem from green plantlet, AL: leaf from albino plantlet, AM: meristem from albino plantlet

The results obtained from the expression analysis by qRT-PCR revealed that all of the evaluated genes appeared to be overexpressed in AL compared to the rest of the tissues evaluated (Fig. 7). This tendency for *PPR* transcripts to be overexpressed in AL is very similar to that previously described in the RNA-seq data (Fig. 6). AM tissue was the only one that showed a slight reduction in the expression of five transcripts (*AaPPR1*, *AaPPR2*, *AaPPR10*, *AaPPR13* and *AaPPR15*) in qRT-PCR results compared to the transcriptomic analysis. In the same context, only the *AaPPR10* and *AaPPR13* showed an overexpression in AL tissue, which had not been identified in RNA-seq data.

### Potential RNA targets for PPR proteins

The bioinformatics strategy based on the construction of RegExp allowed for the identification of hypothetical RNA targets for nine of the twelve chloroplastic PPR proteins in the chloroplast genome of *A. angustifolia* (GenBank-NCBI, accession number MW540498) (Table 1). The RNA targets identified were: *ycf1* for AaPPR1, *rpoC1* and *rps12* for AaPPR2, *trnK-UUU* for AaPPR5, *rps16*, *petN* y *atpE* for AaPPR6, *rpoC1, psbC*, *petA* and *rrn23* for AaPPR10, *ycf2* for AaPPR11, *atpA*, *rps14*, *trnI-GAU* and *ycf1* for AaPPR13, *rbcL* and *rpl33* for AaPPR15 and *ycf2*, *psbD*, *psbC*, *psaB* and *ndhG* for AaPPR18 (Additional file 1: Fig. S7). Nine of the RNA targets encode photosynthesis-related proteins: three are subunits of photosystems I (psaB) and II (psbC and psbD), two are components of the cytochrome b/f complex (petA and petN), two are subunits of ATP synthase (atpA and atpE), one is a subunit of NADPH dehydrogenase (ndhG) and the large subunit of RuBisCO (rbcL). Four of the targets encode ribosomal proteins (rps12, rps14, rps16 and rpl33), two are transfer RNAs (*trnI-GAU* and *trnK-UUU*) and one is a ribosomal RNA (*rrn23*). One target is a subunit of plastid encoded RNA polymerase (PEP) (*rpoC1*). Two of the targets have unknown functions (*ycf1* and *ycf2*). The *rps12*, *rrn23*, *ycf2* and *trnI-GAU* genes are duplicated in the chloroplast genome of *A. angustifolia* and both copies encode the same RNA. For the AaPPR2, AaPPR10, AaPPR11, AaPPR13 and AaPPR18 proteins that have these two RNAs as targets, both were identified in this study. The hypothetical target sequences to which AaPPR2, AaPPR6 and AaPPR13 bind are located in introns of the *rpoC1* and *rps12*, *rps16* and *trnI-GAU* RNAs, respectively. The rest of the evaluated proteins have their targets in exons. In the case of APPR3, APPR4 and AaPPR20, the number of identified targets was very high (with more than 60 RNA targets per protein, data not shown).

**Table 1:** Hypothetical RNA targets of chloroplastic PPR proteins of *A. angustifolia*

Transcript ID	Regular expression	RNA targets	RNA target sequence
*AaPPR1*	U[AU]UUU[AU]U[CAU][AC]U[AU]UU[ACGU][ACGU]	*ycf1* (CDS)	5′-UAUUUUUCAUUUUCA-3’
*AaPPR2*	[ACGU][ACGU][UCG][AU][UCG][UCG]A[UCG]G[UCG][AU]GAU[ACGU]	*rpoC1* (intron)	5′-UCUUUUAGGGAGAUA-3’
		rps12* (intron)	5′-GCUUGGAGGGAGAUC-3’
*AaPPR5*	[ACGU][CG]A[AU]GAAAA[AU]G[ACGU][AU]A[ACGU][GU]	*trnK-UUU* (intron)	5′-AGAAGAAAAUGGAAUU-3’
*AaPPR6*	[UC][GC][GC]A[CU][CA]UAG[GU]	*rps16* (intron)	5′-UCGACAUAGU-3’
		*petN* (CDS)	5′-UGGAUAUAGU-3’
		*atpE* (CDS)	5′-UGGAUAUAGG-3’
*AaPPR10*	[ACGU]C[ACGU]UUCC[ACGU]CC	*rpoC1* (CDS)	5′-UCCUUCCUCC-3’
		*psbC* (CDS)	5′-GCGUUCCCCC-3’
		*petA* (CDS)	5′-UCUUUCCCCC-3’
		rrn23* (exon)	5′-GCGUUCCGCC-3’
*AaPPR11*	[UC][UC][ACGU]A[ACGU]U[ACGU][ACGU][ACGU][ACGU][ACGU][ACGU]C[ACGU][UC]UCUU[CU]U	ycf2* (CDS)	5′-UUCAAUCCUUUUCCUUCUUCU-3’
*AaPPR13*	[GU][ACGU][ACGU]C[ACGU][ACGU]G[ACGU][ACGU][ACGU]AUGU	*atpA* (CDS)	5′-UAUCCAGGAGAUGU-3’
		*rps14* (CDS)	5′-UGGCAAGAAAAUGU-3’
		trnI-GAU* (intron)	5′-UGACCCGGAGAUGU-3’
		*ycf1* (CDS)	5′-UCUCAAGCAUAUGU-3’
*AaPPR15*	UUG[CU]CGC	*rbcL* (CDS)	5′-UUGCCGC-3’
		*rpl33* (CDS)	5′-UUGUCGC-3’
*AaPPR18*	UC[GC]UAU[AU][CU][ACGU]G	ycf2* (CDS)	5′-UCCUAUACGG-3’
		*psbD* (CDS)	5′-UCCUAUUUGG-3’
		*psbC* (CDS)	5′-UCGUAUUCUG-3’
		*psaB* (CDS)	5′-UCGUAUUUGG-3’
		*ndhG* (CDS)	5′-UCCUAUUUUG-3’

*CDS* coding sequence

[]: character group, e.g. [AGCU] match the characters A,G,C and U; asterisk (*): RNAs generated from duplicated genes present in the inverted repeats (IR) regions of the *A. angustifolia* chloroplast genome

## Discussion

The PPR protein family is characterized by presenting a structure made up of a tandem array of PPR motifs [16]. In the present research, we used a wide array of bioinformatic tools such as PPRFinder, HMMER, InterProScan, TPRpred, MEME, and BLASTp to identify putative PPR sequences in somaclonal variants of the non-model plant *A. angustifolia* (Fig. 1)*.* We identified 1581 putative PPR sequences in the green (G) and albino (A) phenotypes (Additional file 2: Table S1), from which 282 were chloroplastic *PPRs* (Fig. 4B). We also evaluated the quantitative expression of 12 *PPRs* (Fig. 7) and their possible targets (Additional file 1: Fig. S7).

PPR proteins are considered central players in plastid RNA metabolism. This is mainly due to the fact that they are very active in the early stages of chloroplast biogenesis, where they play roles as post-transcriptional regulators [15, 16]. We found that chloroplastic *PPR* transcripts in the albino somaclonal variant presented a higher expression in the albino leaf (AL) in comparison with the green leaf (GL) or the meristem tissues (GM and AM) (Figs. 6 and 7). This could suggest a nucleus-chloroplast miscommunication during chloroplast biogenesis [6, 43].

Since PPR proteins are encoded by nuclear genes, their transcription, processing and translation are carried out by the nuclear machinery and finally exported to their organelles of action, either the mitochondria or the chloroplast [44]. The absence of functional chloroplasts in mesophyll cells of the leaf in the *A. angustifolia* albino variant [45] opens new research avenues about the functional role of chloroplastic PPRs in retrograde signaling. *GUN1* (*GENOMES UNCOUPLED 1*), a central regulator of plastid-to-nucleus retrograde signaling, is a *PPR* highly expressed in young and expanding leaves of *A. thaliana*, while in mature leaves, stem, and roots its expression is strongly reduced [46]. This overexpression in young *Arabidopsis* seedlings has been described in other chloroplastic *PPR* genes such as *PDM3* and *AtDPG1* [32, 47]. Therefore, it suggests that the expression of *PPRs* in plants is conditioned by two factors: the state of cellular differentiation and the state of differentiation of the plastid.

Most of the available information on chloroplastic *PPRs* and their functions is from knockout or knockdown mutants in plants [13]. The partial or total reduction in the expression of chloroplastic *PPR* in these mutants has been associated with the emergence of phenotypes with alterations in their pigmentation, dominated by those with albino and pale-green phenotypes [9]. On the other hand, the alterations have also been associated with other changes. For instance, *osppr16* and *ossla4* mutants in *O. sativa* showed damage to the structure of thylakoid membranes, low accumulation of photosynthetic pigments and disruption of photosynthetic capacity and stomatal variables [27, 48]. In *A. thaliana*, the *atppr4* mutant exhibited seedling lethality under autotrophic growth conditions, alterations in key embryo morphogenetic events and defects in plastid protein synthesis [49]. PPR mutants in *Z. mays* such as *emb-7 l* showed reduction of plastid-encoded RNA polymerase (PEP) and increased expression of plastid-encoded RNA polymerase (NEP)-dependent chloroplastic genes, respectively [50]. We found that the expression profiles of chloroplastic *PPRs* in the albino plantlets of *A. angustifolia* were overexpressed (Figs. 6 and 7), unlike the reported pigment-impaired PPR mutants in maize, *Arabidopsis* and rice, in which the expression of chloroplastic *PPR*s is very low or absent [27, 48–50].

Our results indicate that the increase in the expression of chloroplastic *PPRs* in phenotypes with alterations in their pigmentation (Figs. 6 and 7) and with numerous undifferentiated plastids, such as the albino plantlets of *A. angustifolia* [45], could be closely related to the blockage of chloroplast biogenesis. It was recently found that in samples from the base of the leaf in wheat, where proplastids are very numerous, the *PPR* transcripts present their highest peak of activity; this result demonstrates their central role in the early biogenesis of the chloroplast. This role was confirmed when the plastid begins to differentiate and mature, which leads to a decrease in the expression of these transcripts [51]. Furthermore, the transcriptomic analysis of four mutants of *A. thaliana* with different degrees of alteration in their pigmentation, such as *apg2*, *cla1*, *apg3*, and *ch42*, support the relationship between the state of chloroplast biogenesis and the expression of *PPR*s. The *apg2*, *cla1* and *apg3* mutants, which showed a strong reduction in photosynthetic pigments and damage to the chloroplast ultrastructure, overexpressed ten chloroplastic *PPR*s compared to the *ch42* mutant, which showed a less severe phenotype and had only two overexpressed chloroplastic *PPR*s [52].

**Fig. 8:**
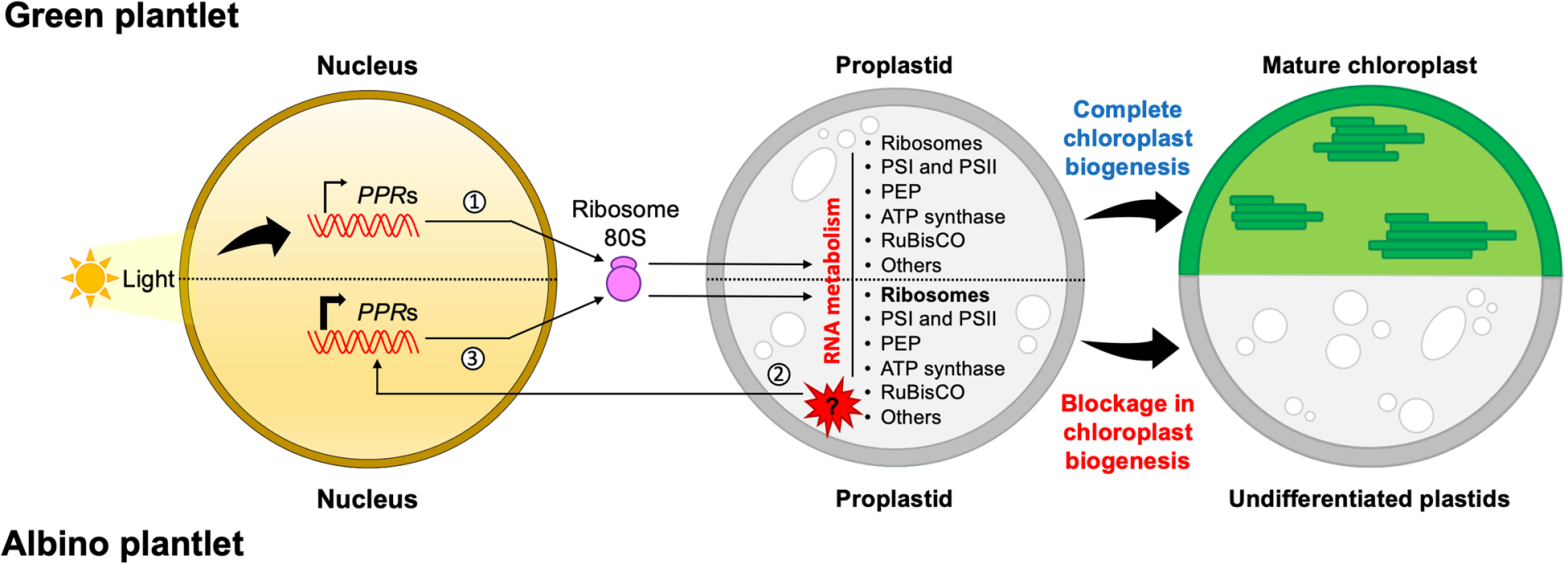
Model of retro-anterograde communication proposed for albino plantlets of *A. angustifolia*. In green plantlets, environmental factors such as light trigger chloroplast biogenesis through the activation of nuclear genes encoding chloroplastic proteins, among which are the *PPR*s (1). This interorganellar nucleus-plastid communication is part of anterograde signaling. *PPR* transcripts must be translated in cytoplasmic ribosomes and subsequently translocated to plastids, where they participate in RNA processing and in the maturation of the proplastids into mature and functional chloroplasts. On the other hand, in the numerous proplastids of the albino *Agave* plantlets, a possible signal of unknown origin (2) seems to activate a retrograde response (plastid-to-nucleus). We propose that when this signal reaches the nucleus, it activates the overexpression of *PPR*s, which is part of a compensatory retro-anterograde response (3) whose objective is to correct the blockage in plastid biogenesis. It is possible that the overexpression of *PPR*s whose targets are RNAs of genes related to translation and the ribosome could play a central role in this corrective action. However, this compensatory response is unable to remove the proplastid from this immature and undifferentiated stage and, therefore, does not reverse the albino phenotype

The high expression levels of *PPR* in the albino plantlets (Figs. 6 and 7) could reveal a key role of these genes during early biogenesis of the plastid, specifically during interorganellar plastid-nucleus communication**.** Recently, a novel interaction involving these two organelles was described in the virescent *cue8* mutant. In the proplastids of this *Arabidopsis* mutant, an unknown retrograde signal triggered a reduction in the expression of sigma factor genes, photosynthesis-associated nuclear genes (PhANGs), and their regulators, and promoted the expression of NEP**.** This resulted in a corrective anterograde response that maintained the replication of the plastome, suppressed the expression of PEP-dependent genes, and retained the plastid in a state of juvenile development, whose maturation process was slower but successful. This process was called corrective retro-anterograde communication [53]. We propose that the high expression of *PPR* could be part of a retro-anterograde compensatory response, very similar to that of the *cue8* mutant. However, this response would fail in the attempt to remove the proplastid from its juvenile developmental stage and reverse the albino phenotype of *Agave* plantlets. At this point, the retrograde signal that activates and maintains *PPR* gene expression in AL is still unknown (Fig. 8). However, the evidence available for the Agave albino variant and other albino variants with this phenotype seems to indicate that this retrograde signal could involve the biosynthetic pathways of tetrapyrroles and carotenoids [54, 55].

One approach that could reveal more information about the importance of the *PPR* genes in this retro-anterograde response focuses on their RNA targets in the plastid. In our search to identify the hypothetical RNA targets of the 12 PPRs using RegExp, targets for nine of them were identified. These targets were related to ribosomes, photosystems, ATP synthase, plastid-encoded RNA polymerase (PEP) and RuBisCO (Additional file 1: Fig. S7). The information available on the RNA targets of orthologous proteins in plant models such as *A. thaliana* reveals that some of the targets identified in *A. angustifolia* are conserved (Table 2) [56–63]. For instance, both AaPPR2 and its orthologous protein OTP81/QED1 have as one of their targets the RNA *rps12*, where OTP81/QED1 exerts C-to-U editing [56]. In the case of AaPPR5 and its orthologous protein SEL1/PDM1, the RNA of the *trnK* gene has been identified as a target, where SEL1/PDM1 participates in splicing [57]. Finally, AaPPR6 and its orthologous protein SVR7 share as a target the mRNA *atpE*, where SVR7 regulates the transcription and translational activation of dicistron *atpB/E* [60]. A particular case is AaPPR10, to which the orthologous protein AmPPR5 binds in *Z. mays* and protects the *trnG-UCC* precursor from the action of endonucleases; this activity indirectly impacts the processing and accumulation of rRNAs and the conformation of the plastid ribosome [61]. This data could be linked to one of the target RNAs of AaPPR10, the RNA of the *rrn23* gene, a finding which reveals a possible site of action that has not been previously identified in other plant models.

**Table 2:** Closest homologues to the nine PPR proteins with identified RNA targets

Transcript ID	RNA targets	Homologs in ***A. thaliana***	PPR subfamily (class)	***E***-value	Identity (%)	RNA targets	References
*AaPPR1*	*ycf1*	AT4G35130	PLS (DYW)	0	51	–	–
*AaPPR2*	*rpoC1, rps12*	AT2G29760 (OTP81/QED1)	PLS (DYW)	0	55	･Editing of *accD*, *matK*, *ndnB*, *rpoB* and *rps12.*	[56]
*AaPPR5*	*trnK-UUU*	AT4G18520 (SEL1/PDM1)	PLS (PLS)	0	54	･Processing of *rpoA.* ･Editing of *accD.* ･Splicing of *ndhA* and *trnK*.	[57–59]
*AaPPR6*	*rps16, petN, atpE*	AT4G16390 (SVR7)	P (SMR)	0	63	･Transcription of ATP synthase subunits *(atpB*/*E, atpH* and *atpF) and psaJ.* *･*Accumulation of ATP synthase subunits *(atpA, atpB, atpE and atpF).* *･Translation rbcL and atpB/E*.	[60]
*AaPPR10*	*rpoC1, psbC, petA, rrn23*	GRMZM2G025409 (ZmPPR5)	P	0	70	･Stabilizing unspliced precursor of *trnG-UCC* by inhibiting an endonucleolytic cleveage event*.* *･*Possible association with *rpl16.* *･*Indirect association with reduction in rRNA processing*.*	[61]
*AaPPR11*	*ycf2*	AT4G30825 (BFA2)	P	0	60	･Barrier to prevent the *atpH/F* transcript degradation by exoribonucleases by binding to the consensus of the *atpF/A* intergenic region	[62]
*AaPPR13*	*atpA, rps14, trnI-GAU, ycf1*	AT5G13770	P	0	50	–	–
*AaPPR15*	*rbcL, rpl33*	AT2G02980 (OTP85)	PLS (DYW)	0	57	･Editing of *ndhD*	[63]
*AaPPR18*	*ycf2, psbD, psbC, psaB, ndhG*	AT4G02750	PLS (DYW)	1.00E-117	45	–	–

The identification of more than one RNA target per protein is not a new event. Currently, PPRs that act on more than one target are known, such as OTP81 / QED1, which performs an editing action on five different sites (*accD*, *matK*, *ndhB*, *rpoB* and *rps12 transcripts*) [56]. Another example is the ZmPPR5 protein that has the RNA of the *trnG-UCC* gene as its main ligand, as well as other ligands with which it is weakly associated, such as the RNA of the *rpl16* gene [61]. A similar case occurs with the ATP4 protein in corn (orthologous of SVR7 in *A. thaliana*), where minor ligands have been reported [19]. The information on the targets of these four *PPRs* (APPR2, APPR5, APPR6 and APPR10) could indicate that the ribosome, and particularly the translation of proteins in the cells of albino leaf tissue, is compromised and, therefore, anterograde corrective action of PPRs may be trying to compensate. However, we cannot discard the possibility that the rest of the RNA targets identified for the PPRs are linked to other protein complexes that act in the plastid and that participate in transcription, photosynthetic metabolism, and ATP synthesis.

## Conclusions

Plants with albino phenotypes exhibit a blockage in chloroplast biogenesis, which positions them as unique and novel models for understanding the mechanisms that regulate nucleus-plastid signaling. Here, we showed that the expression of chloroplastic *PPR*s is dependent on the state of differentiation of the plastid, being higher in the early phases of chloroplast biogenesis; that is, when the proplastid phase dominates. Chloroplastic *PPR* genes in the leaf tissue of albino plantlets exhibited an increased expression because of stagnation of plastid development in leaf mesophyll cells. These results reveal the unexpected finding of high expression levels of chloroplastic *PPRs* in albino plants; this expression could be part of a retro-anterograde communication, where these genes are playing a compensatory function that tries to restore the normal process of development and maturation of proplastids.

## Methods

### Plant materials

In this study, two *A. angustifolia* Haw. somaclonal lines that differed phenotypically from each other were used, obtained by micropropagation from plants with the same genetic background [64, 65]. Plantlets from the phenotypes green (G) and albino (A) (Additional file 1: Fig. S1) were cultured in Magenta boxes containing 50 ml of modified Murashige and Skoog (MS) medium [66] supplemented with 2,4-D (0.11 μM) and 6 BA (22.2 μM) and solidified with agar (0.175%) and gel-rite (0.175%) [67]. The plantlets of each phenotype (G and A) were incubated in a growth chamber at 27 ± 2 °C under a 12-h photoperiod (40 μmol/m^− 2^/s^− 1^). Both leaf and shoot apical meristem (referred to as “meristem” throughout the article) tissues were excised using a scalpel blade from 2.5-cm tall plantlets of each phenotype. This resulted in four study conditions: green leaf (GL), albino leaf (AL), green meristem (GM), and albino meristem (AM). The authors have complied with all relevant institutional and national guidelines and legislation in experimental research and field studies on plants.

### Searching the *A. angustifolia* transcriptome for PPR sequences

The search for PPR sequences was carried out in the *A. angustifolia* transcriptome results (unpublished data) of GL, GM, AL and AM tissues. The nucleotide sequence data of the *PPR*s for this study were deposited at NCBI in the nucleotide database under accession numbers OM156485 - OM158065. To identify the candidate coding regions and the peptide sequences in the assembled transcripts, TransDecoder software (vers. 5.5.0) (https://github.com/TransDecoder/TransDecoder/wiki) was used, set at a minimum ORF ≥31 amino acid residues (AA) [28].

The putative PPR peptide sequences were searched using the software HMMER (vers. 3.3.2) [68] and PPRFinder, Pfam and CDD’s profiles. The tool PPRFinder (vers. 1.0) [20, 28] and the *all_PPR.hmm* profile were used to identify PPR motifs in transcriptomic data using *hmmsearch* option from HMMER with the default parameters. The criteria to identify the sequences with motifs of interest was a cutoff score of 0, for SS motifs a score > 10 and for the DYW motifs a score > 30 [20, 28]. All the sequences with a single motif were discarded except those that contained a complete single DYW functional domain [28, 69]. The other two resources used for the identification of sequences with PPR motifs were Pfam [70] and Conserved Domains Database (CDD, NCBI, July 2020). Six full domain alignments related to PPR sequences were downloaded from the Pfam database [PPR (PF01535), PPR_1 (PF12854), PPR_2 (PF13041), PPR_3 (PF13812), DYW_deaminase (PF14432) and PPR_long (PF17177)], and seven alignments from CDD [one from TIGRFAM (TIGR00756), three from Protein Clusters (PLN03081, PLN03218, PLN03077) and the accession sd00004]. An HMM profile was built for each alignment using HMMER. These profiles were used to search for PPR sequences with an *E*-value cutoff of ≤1e-10. Venn diagrams were constructed to represent the number of ORFs identified using the InteractiVenn digital tool [71].

### Filtering PPR sequences

The recovered sequences were filtered using a sum score cutoff of > 40 with PPRFinder. Only the sequences that contained a consecutive array of PPR motifs in the same strand, regardless of the reading frame (RFs), were conserved. The sequences were joined by adding “X” residues to maintain approximate length and indicate the binding site if structural continuity was observed between two ORFs of different RFs [28]. Although these joined sequences are part of the number of sequences reported and analyzed in this study, due to their hypothetical nature they were not considered in final analyses. Additionally, the criteria reported by [20] were taken into account; these allow the analysis to be more rigorous when identifying PPR motifs.

To contrast and improve the prediction and functional annotation of PPR motifs made by PPRFinder, an analysis was carried out with TPRpred software (vers. 1.0) [72] using the default parameters and a score cutoff greater than or equal to 12, and InterProScan 5 (version 5.45–80.0) [73] using InterPro’s signatures (Pfam, TIGRFAM and PrositeProfiles) to detect probable PPR motifs/domains.

### Sequence analysis with DYW and E+ domains

Sequences with full DYW domain were analyzed using the MEME suite (vers. 5.3.3) [74]. The matrices obtained with MEME were used as a reference to search for conserved regions (PG box, active site, and C-terminal) in the sequences with truncated DYW domain of the E+ class using the FIMO tool with a *p*-value cutoff of ≤1e-5. To visualize the conserved regions in the DYW domain, multiple sequence alignments were performed with the MAFFT tool (vers. 7.471) [75].

### Functional annotation

Ortholog genes of the putative PPR sequences were searched by running a local BLASTp (vers. 2.10.1+) [76], using a filtered file of characterized and high-quality plant PPR sequences downloaded from the RefSeq database (NCBI, March 2021) [77]. The local BLASTp search was executed with the default parameters, and sequence pairwise alignments that showed an identity percentage equal or greater than 50% were selected. For sequence alignments with multiple hits, only the best match was selected and reported. The subcellular localization of PPR sequences was predicted using Predotar (vers. 1.04) [78] and TargetP (vers. 2.0) software [79]. For sequences whose subcellular localization was not possible to determine using this prediction strategy, Blast hits of their orthologs were filtered for the keyword “chloroplast” to identify hypothetical plastid sequences.

### RNA extraction and cDNA synthesis

Expression analyses were performed by quantitative real-time PCR (qRT-PCR). Total RNA from AL, GL, AM and GM tissues was extracted with TRI Reagent® (Sigma-Aldrich) according to the manufacturer’s instructions. After ethanol precipitation, the RNA was resuspended in 30 μL RNA-free water and treated with RNase-free DNase I. The quality of extracted RNA was visualized on native agarose gel at 1.0%. The cDNA was synthesized using oligo (dT)18 with SuperScriptTM IV Reverse Transcriptase (Thermo Fisher Scientific).

### Relative quantification by qRT-PCR

All of the PPR sequences identified were filtered and grouped according to the following criteria: 1) complete structure (exhibited a start codon and a stop codon), 2) a hypothetical chloroplast localization inferred from orthologs of other plant species, and 3) differential expression detected in transcriptomic analysis. Twelve putative *PPR* sequences were chosen to validate their expression by qRT-PCR with members of two subfamilies of the PPR family covering the three criteria described above. *Actin*, *tubulin* and *18S* rRNA were used as reference genes. The oligonucleotide pairs used are listed in Additional file 2: Table S3 [80]. Heatmaps were constructed using ComplexHeatmap software (vers. 2.4.3) [81] to represent the expression profiles of the PPR sequences.

The analysis by qRT-PCR was performed using a Rotor-Gene Q (Qiagen). Three ten-fold serial dilutions (10^− 1^, 10^− 2^ y 10^− 3^) of cDNA from GL tissue were quantified to generate standard curves for each primer pair. The reaction efficiency was calculated based on the slopes of each standard curve. The efficiency of the oligonucleotide pairs was between 90 and 110% as recommended. Each qRT-PCR reaction was performed in a final volume of 20 μL using: 0.25 μL of each primer at 10 μM, 10 μL of PowerUp™ SYBR™ Green Master Mix (2X) (Applied Biosystems), 100 ng of cDNA, and nuclease-free water. The thermocycler program consisted of UDG activation at 50 °C for 2 min, an initial denaturation at 95 °C for 3 min, followed by 35 cycles each with 30 s denaturation at 95 °C, 30 s annealing at 60 °C, 60 s extension at 72 °C, and a final step of extension for 5 min at 72 °C. To analyze, relative expression data was used to perform the 2–ΔΔCT method [82] using three technical replicates. The relative expression data generated by qRT-PCR were subjected to one-way analysis of variance (ANOVA) (*P* ≤ 0.05), and the statistical differences between tissues were obtained by a Tukey post-hoc test (P ≤ 0.05) using RStudio software (vers. 1.4.1106) [83]. The Graph Pad Prism (vers. 9.2.0) (Graph Pad software, www.graphpad.com) was used to design graphs.

### RNA target prediction

To identify the amino acid residues at the 5th and last position for each motif in a PPR protein, PPRFinder [20, 28] and PPRCODE prediction server (vers. 1.6.11) [84] were used in twelve validated chloroplastic *PPR* genes. Regular expressions (RegExp) were constructed from the hypothetical sequences of the RNA targets identified by PPRfinder and PPRCODE and searches were carried out in the genes encoded by the chloroplast genome of *A. angustifolia* (data were downloaded from GenBank-NCBI, accession number MW540498) [85]. The constructed RegExp are described in Table 1.

## Supplementary Information

**Additional file 1: Figure S1.** The green and albino phenotypes of *A. angustifolia* plantlets. Individual green (A) and albino (B) plantlets. The meristematic (GM and AM) and foliar (GL and AL) tissues used in this study are indicated in both plantlets. **Figure S2.** Venn diagram of the putative PPR sequences identified in the *A. angustifolia* transcriptome. The diagram (A) summarizes the 3232 sequences that presented PPR motifs and that were identified using PPRFinder, Pfam and CDD profiles. The diagram (B) shows the number of sequences that presented PPR motifs using TIGRFAM, PROSITE and Pfam profiles and TPRpred software in the 1980 previously filtered PPR sequences. The asterisk (*) indicates the databases that were used as part of the analysis in InterProScan 5. **Figure S3.** Sequence logos for the three regions of the DYW domain identified with MEME. The identification of these regions was carried out using the 231 putative PPR proteins of the DYW class identified in *A. angustifolia*. This domain has a length of ∼136 amino acid residues. (A) Logo of the PG box region with a length of 24 residues that is located between residues 1–26 of the DYW domain. (B) Logo of the region of the active site with a length of 32 amino acids that is located between residues 68–99 of the DYW domain. (C) Logo of the C-terminal region with a length of 25 amino acids that is located between residues 112–126 of the DYW domain. **Figure S4.** Multiple alignment of 232 sequences of the DYW class. Only the three conserved regions of the DYW domain (PG box, active site and C-terminal) are shown in the alignment. The black bars indicate the 23 sequences discarded as exhibiting individual incomplete DYW domains and lacking PPR motifs at the N-terminus. **Figure S5.** Multiple alignment of the 86 sequences of class E+. Only the three conserved regions of the classic DYW domain (PG box, active site and C-terminal) are shown in the alignment. The black bars indicate the 21 sequences that were discarded due to lacking motifs at the N-terminal and the PG box region in the DYW domain. **Figure S6.** Distribution of the number of PPR sequences of *A. angustifolia* with homologues in other species. **Figure S7.** Prediction of the potential RNA targets for nine chloroplastic PPR proteins. Each diagram represents an individual PPR sequence. The sequences were ordered by subfamily: PLS subfamily (A) and P subfamily (B). The motifs identified in each PPR protein are represented by gray rectangles in tandem. The type of motif is indicated at the top of each rectangle. The inferred motifs are indicated with an “i” after the corresponding motif name. The residues at the 5th and last position that determine nucleotide-binding specificity are shown with capital letters. The most probable combinations of nucleotides recognized by each PPR motif are marked in blue letters. Together these combinations represent the hypothetical sequence of the RNA target, and were considered for the design of the RegExp. The potential RNA targets (marked in red letters) as well as its complete nucleotide sequence identified after the search with RegExp are presented at the bottom of the scheme. Question marks (?) indicate there is no information available to identify the PPR code, “X” indicates any RNA nucleotide and asterisks (*) indicate that the RegExp was identified in two copies of the same gene.

**Additional file 2: Table S1.** Structural characteristics of the 1581 putative PPR sequences filtered with the bioinformatic pipeline. **Table S2.** Log2 Fold change values of the twelve chloroplastic PPR transcripts selected from RNA-seq data of of *A. angustifolia*. **Table S3.** Oligonucleotides designed from the twelve PPR transcripts selected for their validation by qRT-PCR.

## Data Availability

The datasets generated and analyzed during the current study are available in the database of the National Center for Biotechnology Information (NCBI) in the nucleotide database under accession numbers from OM156485 to OM158065 (https://www.ncbi.nlm.nih.gov/nuccore/?term=) and included in this published article and its supplementary information files.
