# Supplementary material for: Chloroplastic pentatricopeptide repeat proteins (PPR) in albino plantlets of Agave angustifolia Haw. reveal unexpected behavior
Source: BMC Plant Biol. 2022 Jul 19;22:352. doi: 10.1186/s12870-022-03742-2 (PMC9295523; doi:10.1186/s12870-022-03742-2)
Supplement: Supplementary file 1 — Additional file 1: Figure S1. The green and albino phenotypes of A. angustifolia plantlets. Individual green (A) and albino (B) plantlets. The meristematic (GM and AM) and foliar (GL and AL) tissues used in this study are indicated in both plantlets. Figure S2. Venn diagram of the putative PPR sequences identified in the A. angustifolia transcriptome. The diagram (A) summarizes the 3232 sequences that presented PPR motifs and that were identified using PPRFinder, Pfam and CDD profiles. The diagram (B) shows the number of sequences that presented PPR motifs using TIGRFAM, PROSITE and Pfam profiles and TPRpred software in the 1980 previously filtered PPR sequences. The asterisk (*) indicates the databases that were used as part of the analysis in InterProScan 5. Figure S3. Sequence logos for the three regions of the DYW domain identified with MEME. The identification of these regions was carried out using the 231 putative PPR proteins of the DYW class identified in A. angustifolia. This domain has a length of ∼136 amino acid residues. (A) Logo of the PG box region with a length of 24 residues that is located between residues 1–26 of the DYW domain. (B) Logo of the region of the active site with a length of 32 amino acids that is located between residues 68–99 of the DYW domain. (C) Logo of the C-terminal region with a length of 25 amino acids that is located between residues 112–126 of the DYW domain. Figure S4. Multiple alignment of 232 sequences of the DYW class. Only the three conserved regions of the DYW domain (PG box, active site and C-terminal) are shown in the alignment. The black bars indicate the 23 sequences discarded as exhibiting individual incomplete DYW domains and lacking PPR motifs at the N-terminus. Figure S5. Multiple alignment of the 86 sequences of class E+. Only the three conserved regions of the classic DYW domain (PG box, active site and C-terminal) are shown in the alignment. The black bars indicate the 21 sequences that were discarded due to [file 12870_2022_3742_MOESM1_ESM.pdf]

Additional file 1: Supplementary figures

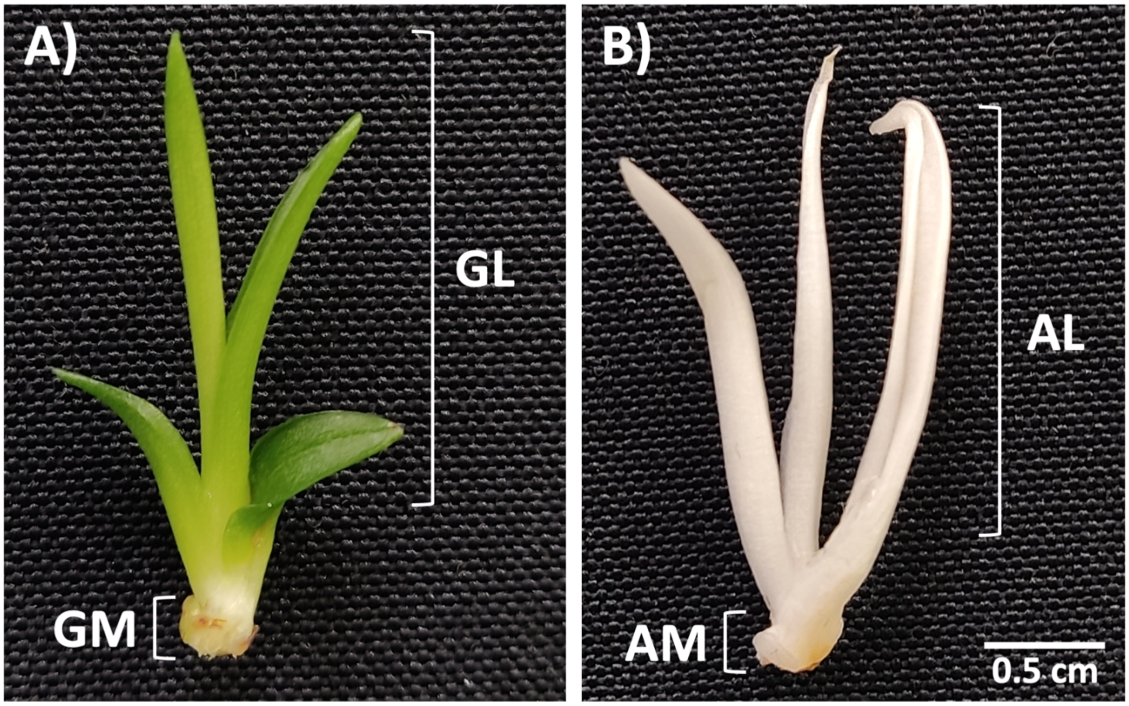

Figure S1. The green and albino phenotypes of *A. angustifolia* plantlets. Individual green (A) and albino (B) plantlets. The meristematic (GM and AM) and foliar (GL and AL) tissues used in this study are indicated in both plantlets.

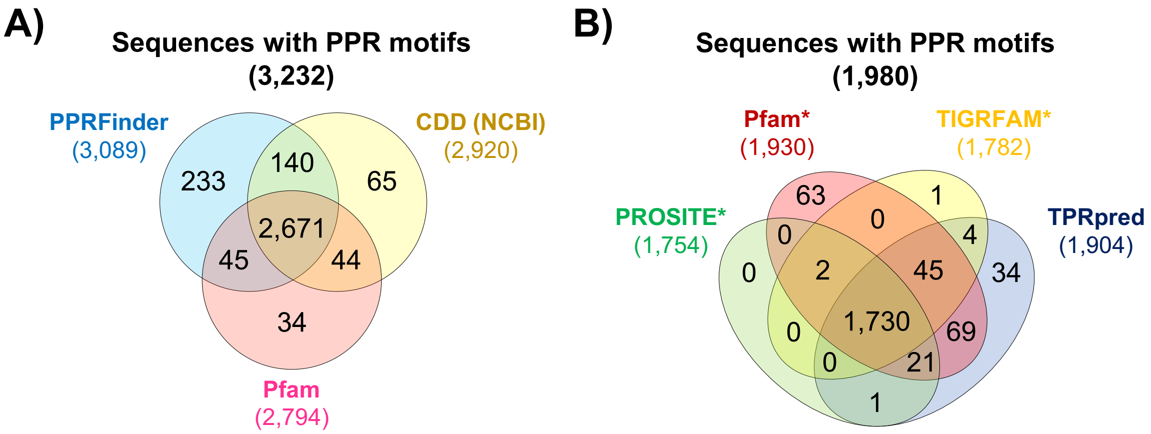

Figure S2. Venn diagram of the putative PPR sequences identified in the *A. angustifolia* transcriptome. The diagram (A) summarizes the 3,232 sequences that presented PPR motifs and that were identified using PPRFinder, Pfam and CDD profiles. The diagram (B) shows the number of sequences that presented PPR motifs using TIGRFAM, PROSITE and Pfam profiles and TPRpred software in the 1,980 previously filtered PPR sequences. The asterisk (*) indicates the databases that were used as part of the analysis in InterProScan 5.

DYW domain

A) PG box (2-25 AA)

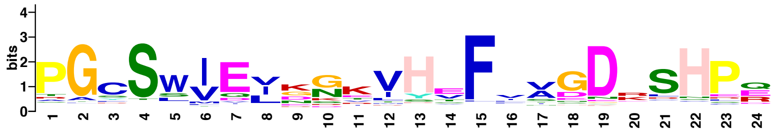

B) Active site (68-99 AA)

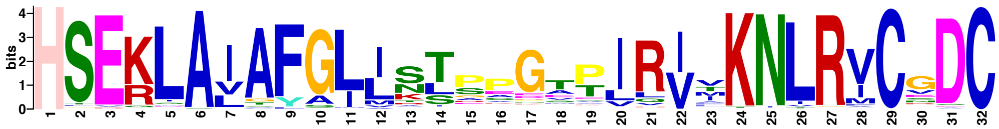

C) C-terminal (DYW) (112-136 AA)

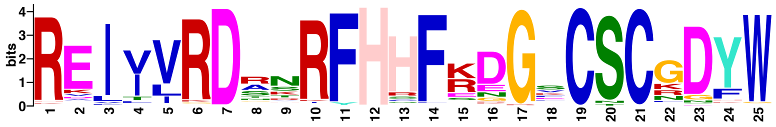

Figure S3. Sequence logos for the three regions of the DYW domain identified with MEME. The identification of these regions was carried out using the 231 putative PPR proteins of the DYW class identified in *A. angustifolia*. This domain has a length of ~136 amino acid residues. (A) Logo of the PG box region with a length of 24 residues that is located between residues 1-26 of the DYW domain. (B) Logo of the region of the active site with a length of 32 amino acids that is located between residues 68-99 of the DYW domain. (C) Logo of the C-terminal region with a length of 25 amino acids that is located between residues 112-126 of the DYW domain.

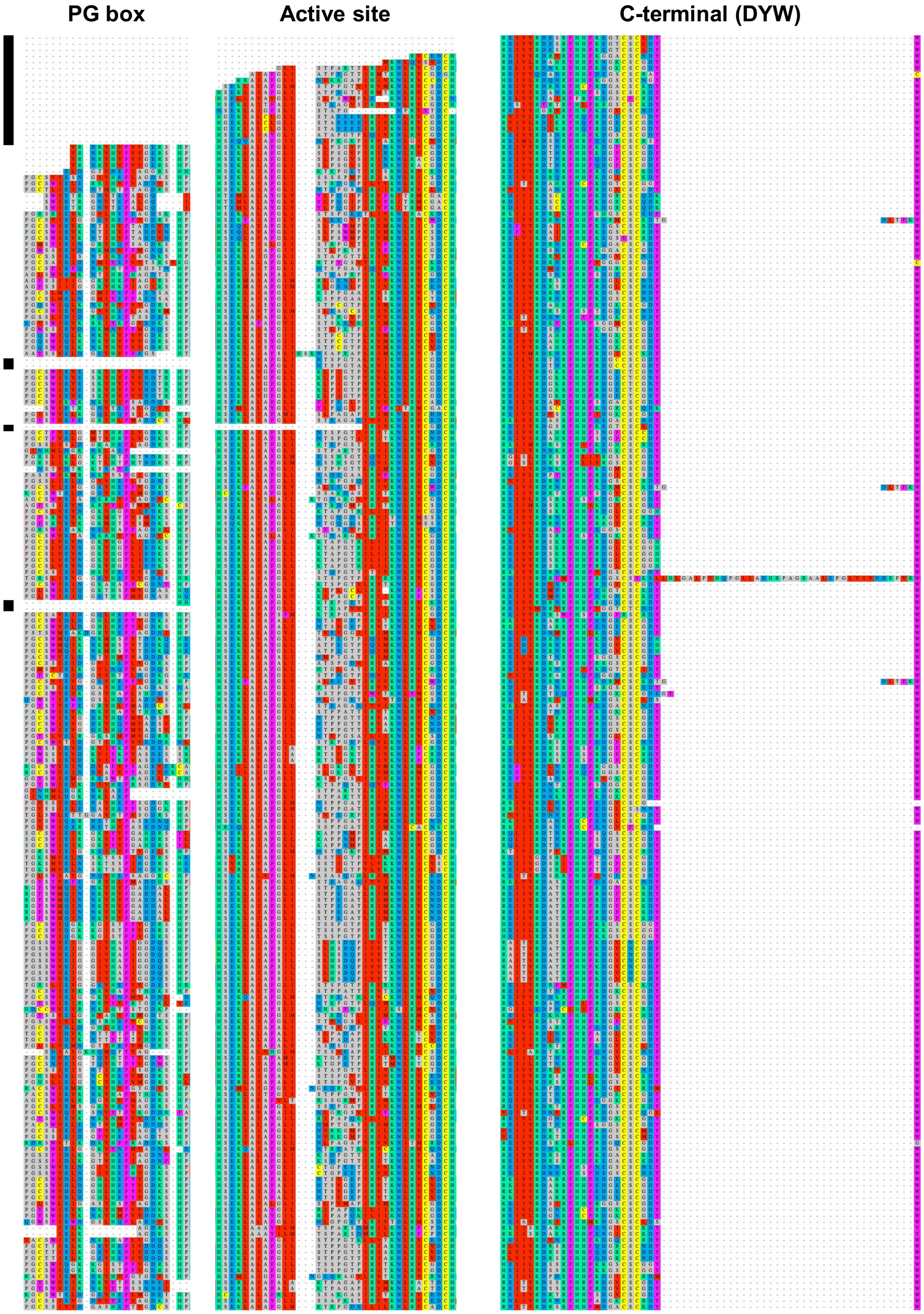

Figure S4. Multiple alignment of 232 sequences of the DYW class. Only the three conserved regions of the DYW domain (PG box, active site and C-terminal) are shown in the alignment. The black bars indicate the 23 sequences discarded as exhibiting individual incomplete DYW domains and lacking PPR motifs at the N-terminus.

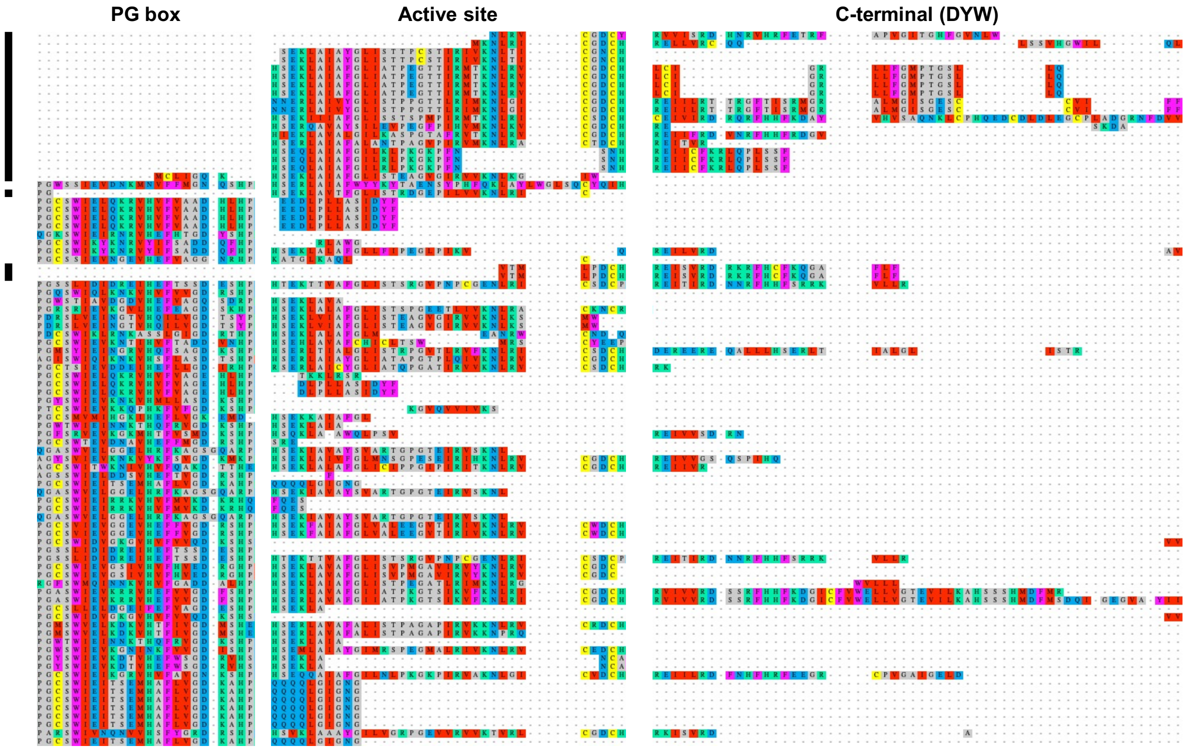

Figure S5. Multiple alignment of the 86 sequences of class E+. Only the three conserved regions of the classic DYW domain (PG box, active site and C-terminal) are shown in the alignment. The black bars indicate the 21 sequences that were discarded due to lacking motifs at the N-terminal and the PG box region in the DYW domain.

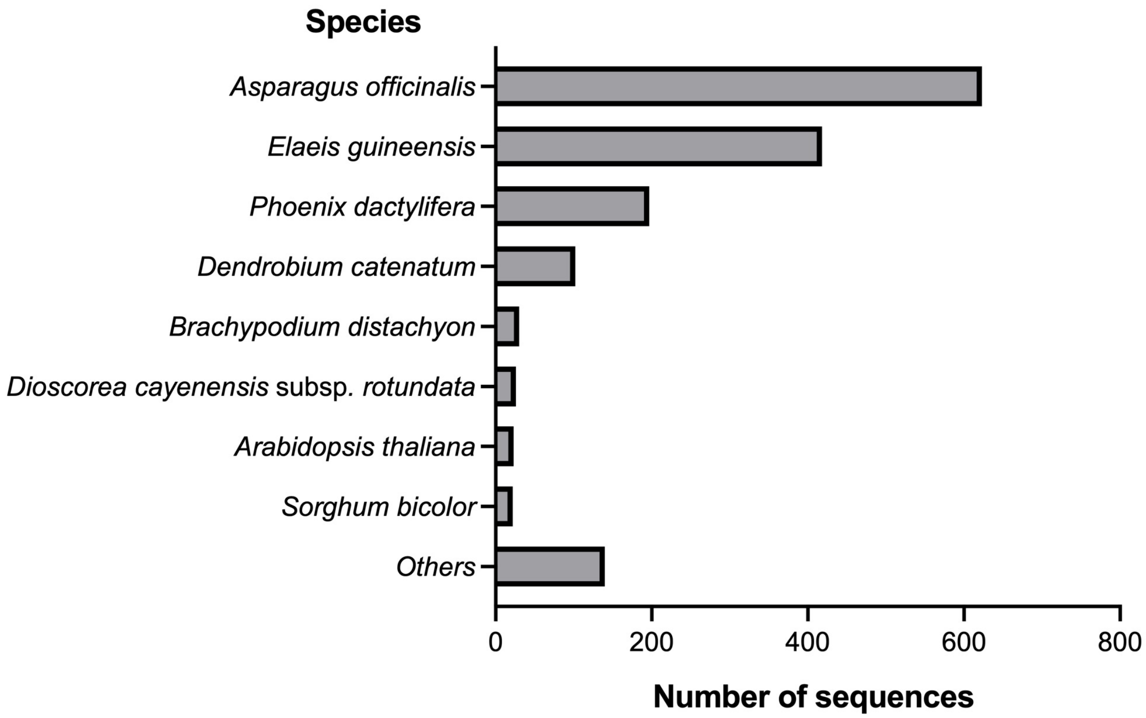

Figure S6. Distribution of the number of PPR sequences of *A. angustifolia* with homologues in other species.

A)

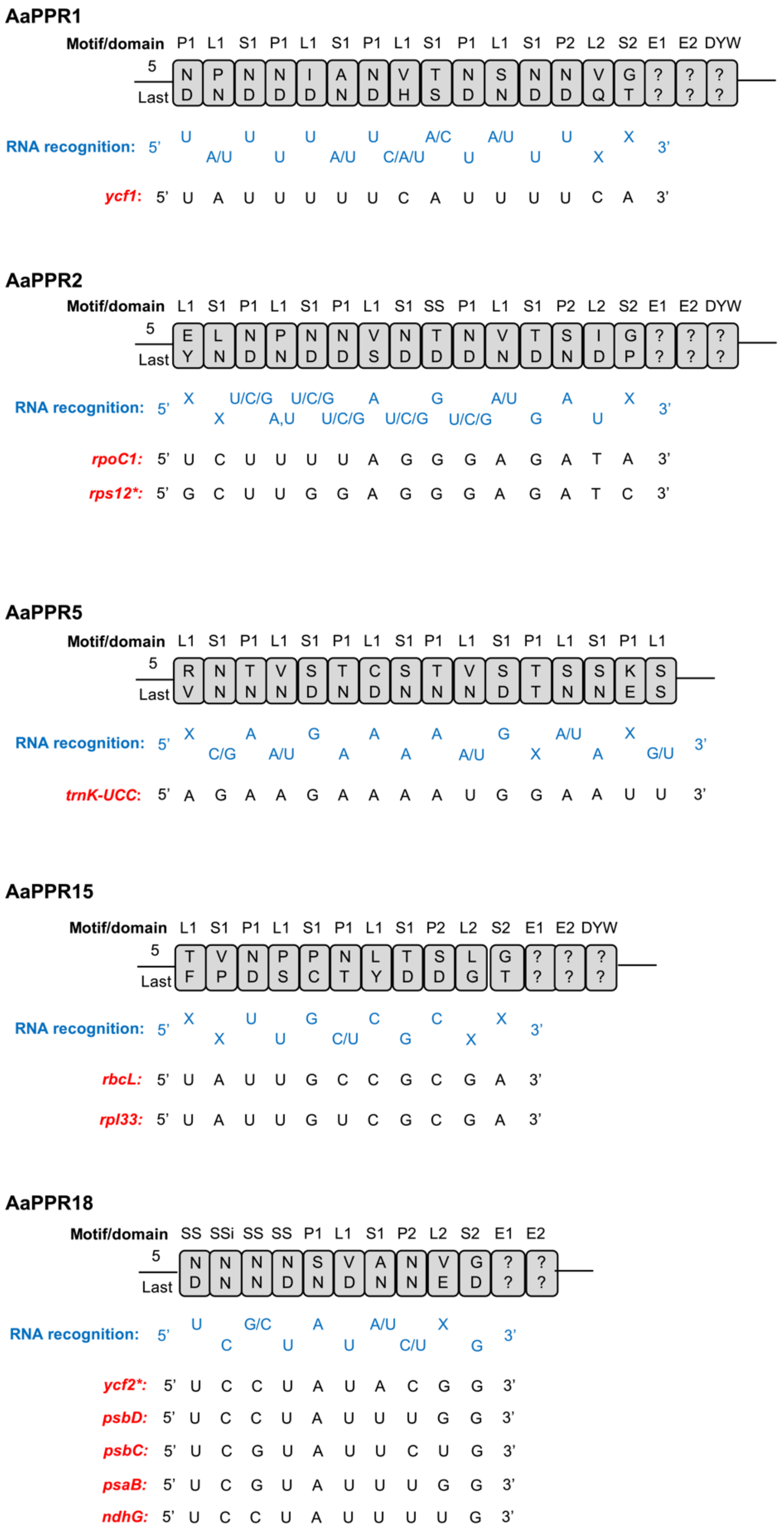
AaPPR1
Motif/domain P1 L1 S1 P1 L1 S1 P1 L1 S1 P1 L1 S1 P2 L2 S2 E1 E2 DYW
5 N P N N I A N V T N S N N V G ? ? ?
Last D N D D D N D H S D N D D Q T ? ? ?
RNA recognition: 5' U A/U U U U A/U U C/A/U A/C U A/U U U X X 3'
ycf1: 5' U A U U U U U C A U U U U C A 3'
AaPPR2
Motif/domain L1 S1 P1 L1 S1 P1 L1 S1 SS P1 L1 S1 P2 L2 S2 E1 E2 DYW
5 E L N P N N V N T N V T S I G ? ? ?
Last Y N D N D D S D D D N D N D P ? ? ?
RNA recognition: 5' X X U/C/G A,U U/C/G U/C/G A U/C/G G U/C/G A/U G A U X 3'
rpoC1: 5' U C U U U U A G G G A G A T A 3'
rps12*: 5' G C U U G G A G G G A G A T C 3'
AaPPR5
Motif/domain L1 S1 P1 L1 S1 P1 L1 S1 P1 L1 S1 P1 L1 S1 P1 L1
5 R N T V S T C S T V S T S S K S
Last V N N N D N D N N N D T N N E S
RNA recognition: 5' X C/G A A/U G A A A A A/U G X A/U A X G/U 3'
trnK-UCC: 5' A G A A G A A A A U G G A A U U 3'
AaPPR15
Motif/domain L1 S1 P1 L1 S1 P1 L1 S1 P2 L2 S2 E1 E2 DYW
5 T V N P P N L T S L G ? ? ?
Last F P D S C T Y D D G T ? ? ?
RNA recognition: 5' X X U U G C/U C G C X X 3'
rbcL: 5' U A U U G C C G C G A 3'
rpl33: 5' U A U U G U C G C G A 3'
AaPPR18
Motif/domain SS SSi SS SS P1 L1 S1 P2 L2 S2 E1 E2
5 N N N N S V A N V G ? ?
Last D N N D N D N N E D ? ?
RNA recognition: 5' U C G/C U A U A/U C/U X G 3'
ycf2*: 5' U C C U A U A C G G 3'
psbD: 5' U C C U A U U U G G 3'
psbC: 5' U C G U A U U C U G 3'
psaB: 5' U C G U A U U U G G 3'
ndhG: 5' U C C U A U U U U G 3'

B)

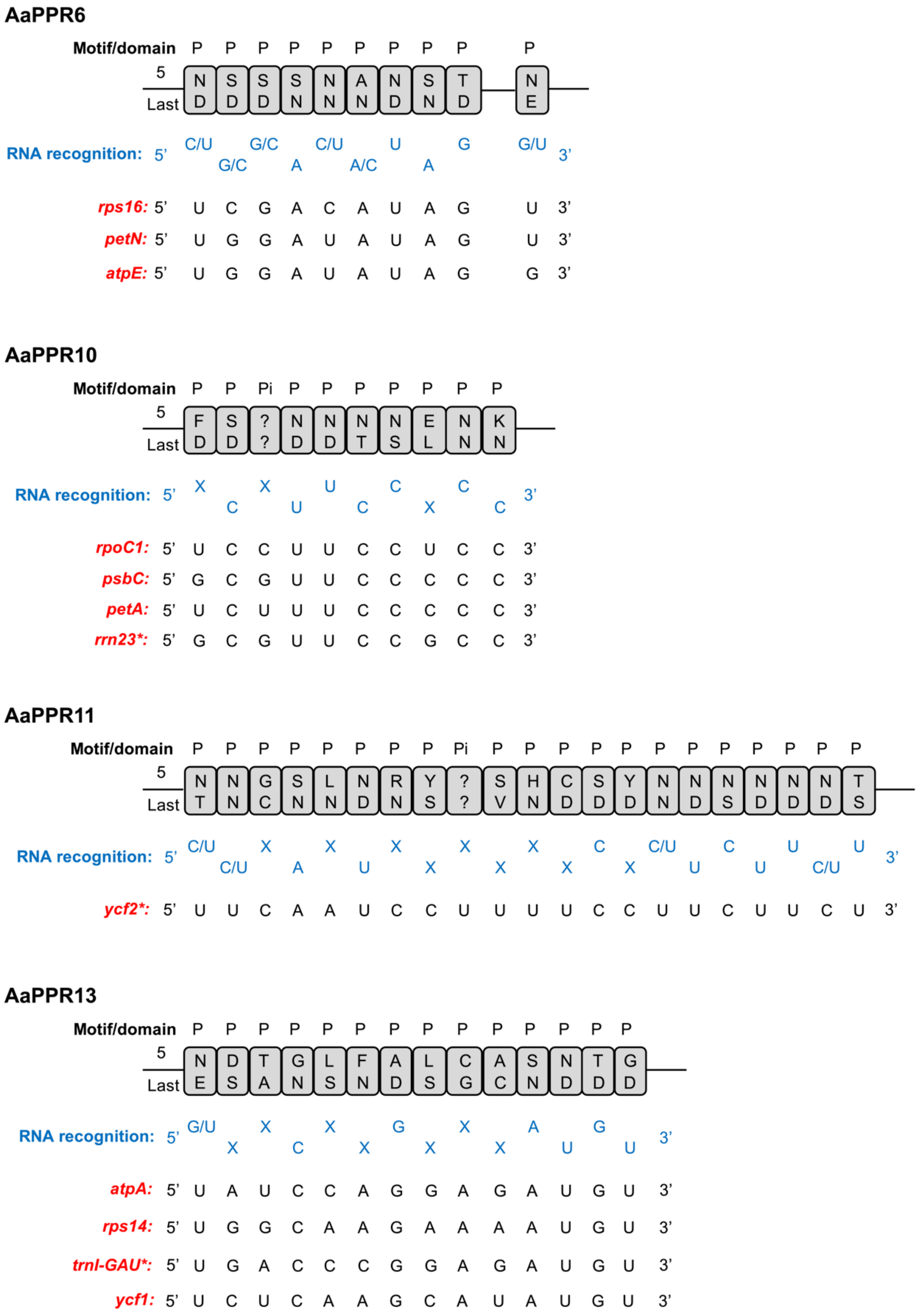
AaPPR6
Motif/domain P P P P P P P P P P
5
Last
N S S S N A N S T N
D D D N N N D N D E
RNA recognition: 5' C/U G/C G/C A C/U A/C U A G G/U 3'
rps16: 5' U C G A C A U A G U 3'
petN: 5' U G G A U A U A G U 3'
atpE: 5' U G G A U A U A G G 3'
AaPPR10
Motif/domain P P Pi P P P P P P P
F S ? N N N N E N K
D D ? D D T S L N N
RNA recognition: 5' X C X U U C C X C C 3'
rpoC1: 5' U C C U U C C U C C 3'
psbC: 5' G C G U U C C C C C 3'
petA: 5' U C U U U C C C C C 3'
rrn23*: 5' G C G U U C C G C C 3'
AaPPR11
Motif/domain P P P P P P P P Pi P P P P P P P P P P P P
N N G S L N R Y ? S H C S Y N N N N N N T
T N C N N D N S ? V N D D D N D S D D D S
RNA recognition: 5' C/U C/U X A X U X X X X X X C X C/U U C U U C/U U 3'
ycf2*: 5' U U C A A U C C U U U U C C U U C U U C U 3'
AaPPR13
Motif/domain P P P P P P P P P P P P P P
N D T G L F A L C A S N T G
E S A N S N D S G C N D D D
RNA recognition: 5' G/U X X C X X G X X X A U G U 3'
atpA: 5' U A U C C A G G A G A U G U 3'
rps14: 5' U G G C A A G A A A A U G U 3'
trnI-GAU*: 5' U G A C C C G G A G A U G U 3'
ycf1: 5' U C U C A A G C A U A U G U 3'

Figure S7. Prediction of the potential RNA targets for nine chloroplastic PPR proteins. Each diagram represents an individual PPR sequence. The sequences were ordered by subfamily: PLS subfamily (A) and P subfamily (B). The motifs identified in each PPR protein are represented by gray rectangles in tandem. The type of motif is indicated at the top of each rectangle. The inferred motifs are indicated with an "i" after the corresponding motif name. The residues at the 5th and last position that determine nucleotide-binding specificity are shown with capital letters. The most probable combinations of nucleotides recognized by each PPR motif are marked in blue letters. Together these combinations represent the hypothetical sequence of the RNA target, and were considered for the design of the RegExp. The potential RNA targets (marked in red letters) as well as its complete nucleotide sequence identified after the search with RegExp are presented at the bottom of the scheme. Question marks (?) indicate there is no information available to identify the PPR code, “X” indicates any RNA nucleotide and asterisks (*) indicate that the RegExp was identified in two copies of the same gene.
